# The impact of living arrangements on dietary patterns among older adults: the mediating effects of loneliness and anxiety

**DOI:** 10.3389/fpubh.2025.1519564

**Published:** 2025-03-12

**Authors:** Yaping Hu, Jiangyan Xiao, Xiaoxiao Li

**Affiliations:** Institute of Population Research, Nanjing University of Posts and Telecommunications, Nanjing, China

**Keywords:** living arrangements, dietary patterns, loneliness, anxiety, older adults, mediation effects

## Abstract

Using data from the 2018 Chinese Longitudinal Healthy Longevity Survey (CLHLS), this study examines the relationship between living arrangements and dietary patterns among older adults aged 60 years and above. Linear regression and quantile regression models were employed to investigate the effects of living arrangements on dietary patterns, while the mediating roles of loneliness and anxiety were tested using the bootstrap method. The empirical results show that, after controlling for demographic and other characteristic variables, living arrangements have a significant positive impact on the dietary patterns of older adults. Compared with those living alone, older adults who live with family (*β* = 0.838) or in institutional settings (*β* = 1.378) exhibit healthier dietary patterns, with the strongest effect observed among those living in institutions. Furthermore, loneliness and anxiety significantly mediate the relationship between living arrangements and dietary patterns, with loneliness exhibiting a stronger mediating effect (*β* = 0.0117) than anxiety (*β* = 0.0037). These findings suggest that living arrangements positively influence older adults’ dietary patterns by reducing loneliness and anxiety. To better implement healthy aging strategies and enhance the overall health of older adults, communities and governments should place greater emphasis on addressing the psychological wellbeing of older adults, providing targeted interventions to optimize their lifestyles and improve their overall health.

## 1 Research background and literature review

As the saying goes, “Food is the paramount necessity of the people.” Human survival and development are inseparable from food intake. In times of resource scarcity, eating was merely a means of survival. However, today, food is endowed with more functions and values. Eating is no longer just about “filling the stomach” (1) but about “living better.” “Better” implies health, and living healthily is a common aspiration among the older adult.

The 14th Five-Year Plan for Healthy Aging proposes guiding older adults to adopt “maintaining physical function and preserving independent living ability” as their health goal. It advocates the awareness that “individuals are the primary person responsible for their health” and reinforces the concept that “families are the first line of defense for health.” The plan aims to encourage older adults and their families to practice healthy lifestyles, promote knowledge on nutrition, physical fitness, and mental health, and educate on preventive measures for sensory, motor, and cognitive function preservation. This initiative seeks to continuously improve older adults’ awareness of core health information and health literacy level (2). Research indicates that diet significantly affects the physiological health of older adults (3). Whether this impact is positive or negative largely depends on the healthiness of dietary patterns (4). Dietary patterns represent a systematic approach to dietary intervention (5). In 2015, the Dietary Guidelines Advisory Committee (DGAC) defined dietary patterns as “the quantities, proportions, variety, or combinations of different foods, beverages, and nutrients (if present) in diets, and the frequency with which they are habitually consumed” (6). Currently, common dietary patterns include the Mediterranean Diet, DASH (Dietary Approaches to Stop Hypertension) Diet, and MIND (Mediterranean-DASH Intervention for Neurodegenerative Delay) Diet (7). Healthy dietary patterns are typically characterized by higher consumption of fruits, vegetables, nuts, legumes, fish, low-fat dairy products, and whole grains, and lower intake of sweets, refined grains, and processed meats (5, 8). In recent years, research on diet has focused primarily on the effects of dietary patterns on population health (8). Studies have shown that healthy dietary patterns are associated with better individual health outcomes, such as reduced risks of all-cause mortality, frailty, cardiovascular diseases, chronic conditions, type 2 diabetes, and neurodegenerative diseases (5, 8–13). Healthy dietary patterns, such as increased consumption of fruits and vegetables, can also lower the risks of depression, anxiety, central obesity, and cognitive health issues like Alzheimer’s disease in older adults (7, 14–17). Conversely, older adults with lower dietary diversity are more likely to experience anxiety or loneliness (18).

At the same time, with the development of society and the economy, significant changes have occurred in the living arrangements of older adults. These changes have had a substantial impact on their dietary patterns. Empirical studies have shown that living arrangements play a critical role in shaping older adults’ dietary habits and nutritional status (19, 20). Older adults living alone or apart from their children are more likely to adopt unhealthy dietary patterns, such as insufficient fruit and vegetable intake and excessive consumption of high-salt and high-fat foods (21–23). Conversely, those living with a spouse or family members often benefit from more regular meals and a greater variety of foods (20, 24). This improvement may be attributed to the mutual support and social interaction among family members, which help enhance dietary quality and nutritional status (20, 25). In addition to the influence of living arrangements on dietary patterns, negative emotions such as loneliness and anxiety are also significant factors affecting older adults’ dietary behaviors (26, 27). Research has found that loneliness may lead to a reduction in food intake among older adults, while anxiety may cause them to choose unhealthy dietary options to cope with psychological stress (28). To explore whether living arrangements influence the dietary patterns of older adults through loneliness and anxiety, previous studies have suggested that anxiety indirectly worsens older adults’ health by affecting lifestyle factors, such as reducing the consumption of healthy foods (29). Moreover, living arrangements indirectly influence older adults’ dietary patterns by alleviating feelings of loneliness and anxiety, further highlighting the importance of family environments (30). This study uses data from the 2018 Chinese Longitudinal Healthy Longevity Survey (CLHLS), focusing on individuals aged 60 years and older. By employing linear regression and quantile regression models, this research investigates the relationship between living arrangements and dietary patterns. Furthermore, the bootstrap method is applied to examine the mediating roles of loneliness and anxiety in this relationship. The study aims to enrich research in this field and provide policy recommendations for improving the health of older adults.

Research has shown that the impact of living arrangements on dietary patterns varies across different age groups. For example, among young adults, those living alone tend to consume higher amounts of carbohydrates (23), likely due to irregular meal times and frequent dining out (31, 32). Additionally, the lack of family support may lead individuals living alone to adopt unhealthy dietary patterns, such as consuming more high-sugar and high-fat foods (31, 32). For older adults, living with children is typically associated with better mental health and nutritional status, while those living alone are more prone to loneliness, depressive symptoms, and malnutrition (20, 33). This highlights the critical role of intergenerational relationships in determining dietary quality (34). Older adults who live alone often exhibit poorer dietary behaviors, including lower intake of grains, fruits and vegetables, meat, eggs, and dairy products (35, 36). Consequently, their overall dietary quality is generally inferior to that of older adults who live with a spouse or children (37). In addition, institutional living has been shown to positively influence the dietary patterns of older adults. Studies indicate that the dining environment, meal arrangements, and flexibility in food choices in long-term care facilities significantly affect older adults’ appetite and overall dietary intake. For instance, a “family-style” dining approach in institutions has been found to increase energy intake and improve overall nutritional status (38). Beyond the impact of living arrangements, psychological factors such as loneliness and anxiety also play a crucial role in shaping dietary patterns among older adults. Previous studies have shown that loneliness affects various aspects of older adults’ lives, including mental health, quality of life (39, 40), dietary patterns, and physical health. International scholars have explored a range of factors influencing older adults’ dietary patterns, including age (41), education level (42), marital status (43), economic conditions (13), and loneliness (44). Loneliness, defined as a discrepancy between actual and desired social relationships (45), has been found to influence dietary choices and behaviors, including food types and nutritional intake (46). In older adults, loneliness contributes to poor nutritional habits and dietary intake and is a significant predictor of anorexia, malnutrition risk, and malnutrition itself (47, 48). Anxiety, one of the most common mental health issues, is highly prevalent among older adults. The prevalence of anxiety disorders in older adults is twice as high as that of affective disorders and four to eight times that of major depressive episodes (49, 50). In China, the prevalence of anxiety disorders among older adults was estimated at 6.79% in 2014, with anxiety symptoms affecting approximately 22.11% of older adults. This rate is significantly higher than other psychological disorders, such as affective disorders or depressive symptoms, and is even higher in medical settings (51). Given current trends, these numbers are likely to have increased. Anxiety can make it difficult for older adults to concentrate, cause dizziness or fainting, and lead to nausea or diarrhea, further weakening their physical state (52). From a macro perspective, the stress caused by anxiety contributes to the global burden of disease, increases demand for healthcare services, and raises mortality rates (53, 54). On an individual level, anxiety leads to reduced quality of life and wellbeing and can result in more severe physical and mental health disorders (55, 56). Among older adults, anxiety is a risk factor for accelerated cognitive decline (57). Anxiety also influences dietary patterns among older adults. Studies suggest that healthier dietary patterns are associated with reduced likelihoods of anxiety or depressive disorders (58). Older adults with greater dietary diversity are less likely to experience anxiety (59). Consequently, there is increasing attention on the effects of anxiety on older adults’ health and health-related lifestyles. In research on cardiovascular patients, Bonnet et al. found that anxiety and depression levels were positively correlated with unhealthy lifestyles. Both anxiety and depression were identified as independent determinants of unhealthy lifestyles in both men and women. Patients exhibiting anxiety or depressive symptoms were more likely to adopt poor dietary habits compared to those without such symptoms (60).

Although the academic research on dietary patterns among older adults is relatively extensive, most studies have been conducted in developed countries where dietary cultures differ significantly from that of China, raising questions about the generalizability of their findings. Furthermore, few studies have explored whether the differential impact of living arrangements on the dietary patterns of older adults is indirectly mediated by loneliness and anxiety. To address these gaps, this study employs linear regression and quantile regression models to examine the effects of living arrangements on the dietary patterns of older adults in China. Additionally, the bootstrap method is used to test the mediating roles of loneliness and anxiety in the relationship between living arrangements and dietary patterns.

## 2 Data, variables, and methods

### 2.1 Data source

The data used in this study were obtained from the Chinese Longitudinal Healthy Longevity Survey (CLHLS). CLHLS is a long-term follow-up survey of older adult individuals organized by the Center for Healthy Aging and Development Studies/National School of Development at Peking University. Following the baseline survey conducted in 1998, follow-up surveys were carried out in 2000, 2002, 2005, 2008–2009, 2011–2012, 2014, and 2017–2018. The most recent survey (2017–2018) covered 15,874 older adult individuals aged 65 and above and collected data on 2,226 older adult individuals who passed away between 2014 and 2018. The data encompass 23 provinces, autonomous regions, and municipalities in China, representing approximately 85% of the total population, and include individuals aged 65 and above as well as their adult children aged 35–64. The project utilized two types of questionnaires: one for surviving older adult individuals and one for family members of deceased older adult individuals. The CLHLS dataset provides extensive and comprehensive information, including data on macroeconomic environments, individual health, and demographic and socioeconomic conditions. It also offers rich data on medical examinations, mortality, biomedical indicators, and genetics, making it an ideal and highly suitable source for studying aging and health issues. For example, Zheng Yang used this dataset to explore the factors influencing health inequality among the older adult (61). Similarly, Song Liangjun and colleagues employed CLHLS data to reveal that the quality of life of Chinese older adult individuals is not only influenced by micro-level factors such as education, mental health, and physical health but also by macro-level factors, including the availability of medical services in the community and the medical supply in their prefecture-level cities (62). This study uses data from the 2018 CLHLS survey of surviving older adult individuals. The questionnaire for surviving older adult individuals includes information on basic conditions of the older adult and their families, social and economic backgrounds, family structure, physical health, and psychological health. The total sample size for the 2018 CLHLS survey was 15,874. For the purposes of this study, the dataset was cleaned as follows: first, individuals under the age of 60 were excluded; second, respondents who were unable to answer during the survey and cases with missing values were removed. After data cleaning, the final valid sample size comprised 6,223 observations.

### 2.2 Variable selection and measurement

#### 2.2.1 Dependent variable: dietary patterns

The dependent variable in this study is dietary patterns. Drawing on previous research (63), the variable is constructed based on responses to three questions from the questionnaire for surviving older adult individuals: (1) “Do you frequently eat fresh fruits?” (2) “Do you frequently eat fresh vegetables?” and (3) “How frequently do you consume the following foods?” The third question includes responses on the frequency of consuming 13 types of foods. From these, the study selects seven specific foods: fish, soy products, tea, garlic, eggs, sugar, and salted or pickled vegetables. Together with the responses to the first two questions, the study measures the frequency of consumption for a total of nine food items. The dietary patterns are assessed based on the respondents’ reported frequency of consuming the following nine food items: fruits, vegetables, fish and other aquatic products, soy products, tea, garlic, eggs, sugar or sweets, and salted or pickled vegetables. The response options for these questions include: “always,” “frequently,” “sometimes,” “rarely,” and “never.” For sugar or sweets and salted or pickled vegetables, responses are scored as follows: “always or almost daily” = 0 points; “sometimes or occasionally” = 1 point; and “rarely or never” = 2 points. For the other seven food items, the scoring is reversed: “always or almost daily” = 2 points; “sometimes or occasionally” = 1 point; and “rarely or never” = 0 points. The total score is calculated by summing the points for all nine food items, resulting in a range of 0 to 18. A higher score indicates a higher frequency of consumption of healthy foods such as fruits, vegetables, fish, soy products, tea, garlic, and eggs, and a lower frequency of consumption of less healthy foods such as sugar or sweets and salted or pickled vegetables. Based on previous studies, we consider a higher score to indicate a healthier dietary pattern (17).

#### 2.2.2 Independent variable: living arrangements

The independent variable in this study is living arrangements. This variable is determined based on responses to the question in the survey: “What is your preferred living arrangement?” Responses are coded as follows: living alone = 0, living with family = 2, and living in an institution = 3.

#### 2.2.3 Mediating variables: loneliness and anxiety

##### 2.2.3.1 Loneliness

The level of loneliness is measured based on the self-assessment question in the surviving older adult questionnaire: “Do you often feel lonely?” Response options include “always,” “frequently,” “sometimes,” “rarely,” and “never,” which are coded as 1, 2, 3, 4, and 5, respectively.

##### 2.2.3.2 Anxiety

The CLHLS survey includes a dedicated anxiety scale to measure the level of anxiety among older adult individuals. Following previous studies (64), respondents were asked about the frequency of seven symptoms experienced in the past 2 weeks, including feeling uneasy and restless, being unable to control worry, excessive worry about various matters, feeling tense, being too restless to sit still, becoming easily annoyed or irritable, and other related symptoms. The response options for each symptom are “not at all,” “several days,” “more than half the days,” and “nearly every day,” which are assigned scores of 4, 3, 2, and 1, respectively. The scores for all seven items are summed to yield a total score ranging from 7 to 28, with higher scores indicating lower levels of anxiety.

#### 2.2.4 Control variables

Based on previous studies (35, 36, 63, 65), the control variables in this study are categorized into three groups: (1) Sociodemographic Characteristics: This includes ethnicity, gender, age, place of residence, and marital status. Ethnicity is recoded as Han and ethnic minorities. Age is recoded into two groups: younger older adult (79 years and below) and older adult (80 years and above). Place of residence is coded as rural or urban. Marital status is coded as “married and living with a spouse” and “separated/divorced/unmarried/widowed.” (2) Socioeconomic Characteristics: This includes education level, occupation before age 60, sufficiency of income, and health insurance participation. Education level is recoded as “no schooling (0 years),” “primary school (1–6 years),” and “secondary school or above (7 years or more).” Occupation before age 60 is coded as agricultural or non-agricultural. (3) Health Behavior: This is measured by whether the individual engages in regular physical exercise. These variables are incorporated into the analysis to control for potential confounding effects and provide a more accurate understanding of the relationship between loneliness, anxiety, and dietary patterns among the older adult (see Table 1).

**Table 1:** Variables and assignments.

Variable category	Variable name	Coding description
Independent variable	Living arrangement	0 = Living alone; 1 = Living with family; 2 = Living in an institution
Dependent variable	Dietary pattern	0–18 points; higher scores indicate healthier dietary patterns
Mediating variables	Loneliness	1 = Always; 2 = Frequently; 3 = Sometimes; 4 = Rarely; 5 = Never
	Anxiety	7–28 points; higher scores indicate lower levels of anxiety
Control variables		
Sociodemographic characteristics	Ethnicity	0 = Ethnic minorities; 1 = Han
	Gender	0 = Female; 1 = Male
	Age	0 = Younger older adult (79 years and below); 1 = Older adult (80 years and above)
	Place of residence	0 = Rural; 1 = Urban
	Marital status	0 = Separated/Divorced/Unmarried/Widowed; 1 = Married and living with spouse
Socioeconomic characteristics	Education level	0 = No schooling; 1 = Primary school; 2 = Secondary school and above
	Sufficiency of income	0 = Insufficient; 1 = Sufficient
	Occupation before age 60	0 = Agricultural; 1 = Non-agricultural
	Health insurance participation	0 = No; 1 = Yes
Health behavior	Physical exercise	0 = No; 1 = Yes

#### 2.2.5 Analytical methods

This study utilized SPSS 26.0 statistical software to conduct data analysis, which proceeded as follows: First, a descriptive statistical analysis was performed to understand the characteristics of the variables used in this study. Subsequently, linear regression models were employed to examine the relationships between living arrangements, other individual characteristic variables, and dietary patterns among the older adult. To address the limitations of linear regression models, quantile regression models were applied to further validate the robustness of the results, following previous research (66). Finally, given the superiority of the Bootstrap method over other mediation testing approaches (67), this study utilized the Bootstrap technique within PROCESS 3.5 to assess mediation effects. The Bootstrap method is a non-parametric approach that estimates indirect effects by resampling from the original sample. If the 95% confidence interval (CI) derived from 5,000 Bootstrap samples does not include zero, the indirect effect is considered significant (35, 36). Furthermore, the study utilizes the *R*^2^ effect size described in previous research to quantify the variance explained by the mediation effects (68).

## 3 Empirical results analysis

### 3.1 Description of sample characteristics

Table 2 presents the descriptive statistical analysis results for the study variables. From the table, we can see that the maximum value for dietary patterns is 18, the minimum value is 0, and the mean is 10.724, indicating that older adult individuals in the sample generally have higher dietary scores and their overall dietary patterns are relatively healthy. The minimum value for anxiety is 7, the maximum is 28, and the mean is 26.388, suggesting that the anxiety levels among older adult individuals in the sample are generally low. In addition, among the 6,223 participants, 154 older adult individuals (2.5%) often feel lonely, while 2,298 older adult individuals (36.9%) never feel lonely, indicating that the overall psychological wellbeing of the older adult population in China is relatively optimistic. Of the sample, 5,818 older adult individuals (93.5%) are of Han ethnicity, and 405 (6.5%) are from ethnic minorities, which is consistent with the ethnic structure of China. There are 2,372 males (38.1%) and 3,851 females (61.9%) in the sample. The older adult in the sample are predominantly older adults (63.5%), and the majority live with family members (80.1%), which aligns with China’s strong family values and the relatively insufficient provision of institutional care. Additionally, the educational level of the older adult is generally low, with only 1.7% having completed secondary education or higher. Furthermore, the majority of older adult individuals (77.7%) worked in agriculture before the age of 60, and more than half of the older adult (63.4%) have not participated in health insurance, although most report that their income is sufficient for living (84.2%).

**Table 2:** Descriptive statistics of variables (*N* = 6,223).

Variable	Category/min value	Frequency/max value	Percentage (%) /mean
Dietary pattern	0.00	18.00	10.724
Anxiety	7.00	28.00	26.388
Living arrangement	Alone	1,093	17.6
	With family	4,987	80.1
	Institutional living	143	2.3
Loneliness	Always	154	2.5
	Often	361	5.8
	Sometimes	1,249	20.1
	Rarely	2,161	34.7
	Never	2,298	36.9
Ethnicity	Minority	405	6.5
	Han	5,818	93.5
Gender	Female	3,851	61.9
	Male	2,372	38.1
Age	Younger group	2,272	36.5
	Older group	3,951	63.5
Residence	Rural	3,186	51.2
	Urban	3,037	48.8
Marital status	Separated/Divorced/Unmarried/Widowed	3,708	59.6
	Married and cohabiting	2,515	40.4
Education level	No formal education	3,693	59.3
	Primary school	2,423	38.9
	Secondary or above	108	1.7
Sufficient income	No	982	15.8
	Yes	5,241	84.2
Occupation before 60	Agricultural	4,836	77.7
	Non-agricultural	1,387	22.3
Health insurance	No	3,946	63.4
	Yes	2,277	36.6
Exercise	No	4,484	72.1
	Yes	1,739	27.9

Dietary pattern and anxiety are numerical variables, with descriptive analysis presenting their minimum values, maximum values, and means. The remaining variables are categorical, and their descriptive analysis presents categories, frequencies, and percentages.

### 3.2 Results analysis

#### 3.2.1 Multicollinearity test

Variance Inflation Factor (VIF) is one of the methods used to detect multicollinearity among variables in the model. Generally, a VIF value ≥5 indicates significant multicollinearity, while a VIF value ≥10 suggests a high degree of multicollinearity (69, 70). As shown in Table 3, all the independent variables in this study have VIF values <5, indicating that the model does not suffer from multicollinearity issues and is well-constructed.

**Table 3:** Results of the multicollinearity test (*N* = 6,223).

Model	Unstandardized coefficients		Standardized coefficients	*t*	Significance	Collinearity statistics	
	B	Standard error	Beta			Tolerance	VIF
(Constant)	6.774	0.370	/	18.325	0.000	/	/
Living arrangement	0.732	0.088	0.103	8.338	0.000	0.974	1.027
Ethnicity	0.159	0.148	0.013	1.076	0.282	0.990	1.010
Gender	0.372	0.081	0.060	4.587	0.000	0.850	1.176
Age	−0.512	0.081	−0.082	−6.309	0.000	0.861	1.162
Place of residence	0.129	0.074	0.022	1.750	0.080	0.964	1.038
Marital status	−0.075	0.075	−0.012	−1.007	0.314	0.977	1.023
Education level	0.570	0.079	0.101	7.193	0.000	0.751	1.331
Sufficiency of income	0.553	0.103	0.067	5.392	0.000	0.941	1.063
Occupation before age 60	0.859	0.090	0.120	9.599	0.000	0.948	1.054
Health insurance participation	−0.208	0.076	−0.034	−2.745	0.006	0.987	1.013
Physical exercise	0.149	0.082	0.022	1.819	0.069	0.976	1.024

^a^Indicates statistical significance at *p* < 0.05. The model reports unstandardized regression coefficients with standard errors in parentheses. Significance levels: *p < 0.05, **p < 0.01, ***p < 0.001.

#### 3.2.2 Model testing

This section uses dietary patterns as the dependent variable, living arrangements as the independent variable, and ethnicity, gender, age, and residence type as control variables to establish a linear regression equation and quantile regression model, as shown in Tables 4, 5.

**Table 4:** Results of the linear regression model (*N* = 6,223).

Variable	Model 1	Model 2	Model 3
Living alone		0(.)	0(.)
Living with family		0.965*** (0.099)	0.838*** (0.097)
Living in an institution		0.569*** (0.264)	0.378*** (0.258)
Ethnicity	0.131 (0.149)		0.175 (0.149)
Gender	0.433*** (0.082)		0.410*** (0.081)
Age	−0.537*** (0.082)		−0.501*** (0.082)
Place of residence	0.142 (0.075)		0.124 (0.074)
Marital status	−0.088 (0.076)		−0.081 (0.075)
Education level	0.613*** (0.080)		0.608*** (0.080)
Sufficiency of income	0.753*** (0.101)		0.740*** (0.101)
Occupation before age 60	0.871*** (0.090)		0.846*** (0.090)
Health insurance participation	−0.212*** (0.077)		−0.212*** (0.076)
Physical exercise	0.153 (0.083)		0.151 (0.082)
_cons	9.690*** (0.184)	9.914*** (0.090)	8.957*** (0.202)
Adjusted R^2^	0.063	0.016	0.075

The models report unstandardized regression coefficients. The values outside the parentheses are unstandardized coefficients (β), and the values inside the parentheses are standard errors. Significance levels: *p < 0.05, **p < 0.01, ***p < 0.001. The living arrangement variable is treated as a dummy variable with “living alone” as the reference group. Bold values denote statistical significance at *p* < 0.01.

**Table 5:** Results of the quantile regression model (*N* = 6,223).

Parameter	*q* = 0.25	*q* = 0.5	*q* = 0.75
Living alone	0(.)	0(.)	0(.)
Living with family	1.000*** (0.200)	1.316*** (0.133)	0.750*** (0.100)
Living in an institution	2.000*** (0.534)	1.737*** (0.355)	1.000*** (0.267)
Ethnicity	1.958E-15 (0.308)	0.316 (0.205)	0.250 (0.154)
Gender	0.500*** (0.168)	0.421*** (0.112)	0.250*** (0.084)
Age	−0.500*** (0.169)	−0.474*** (0.112)	−0.500*** (0.085)
Place of residence	1.885E-15 (0.154)	0.053 (0.103)	0.250*** (0.077)
Marital status	−6.238E-16 (0.156)	−0.053 (0.104)	−2.788E−15 (0.078)
Education level	0.500*** (0.165)	0.526*** (0.110)	0.500*** (0.082)
Sufficiency of income	1.000*** (0.209)	0.947*** (0.139)	0.500*** (0.104)
Occupation before age 60	1.000*** (0.187)	0.947*** (0.124)	0.750*** (0.093)
Health insurance participation	-1.995E-15 (0.158)	−0.105 (0.105)	−0.250*** (0.078)
Physical exercise	1.154E-15 (0.171)	0.368*** (0.113)	0.250 (0.085)
Pseudo R^2^	0.038	0.033	0.027

The models report unstandardized regression coefficients. The values before the parentheses are unstandardized coefficients, and the values within the parentheses are standard errors. Significance levels: *p < 0.05, **p < 0.01, ***p < 0.001. The living arrangement variable is treated as a dummy variable, with “living alone” as the reference group. Bold values denote statistical significance at *p* < 0.01.

### 3.3 Linear regression model

Table 4 presents the linear regression results for dietary patterns among the older adult. In Table 4, Model 1 includes all control variables, Model 2 adds the independent variable to Model 1, and Model 3 incorporates both the independent variable and all control variables. The explanatory power of the models gradually improves, with the adjusted R^2^ increasing from 0.063 in Model 1 to 0.075 in Model 3. Overall, living arrangements have a significant positive impact on dietary patterns among the older adult. Compared to those living alone, older adult individuals living with family (*β* = 0.838) or in institutions (*β* = 1.378) exhibit healthier dietary patterns, with the strongest effect observed among those living in institutions. Additionally, gender shows a significant positive correlation with dietary patterns, with male older adult individuals exhibiting healthier dietary patterns than their female counterparts. Age, on the other hand, is significantly negatively correlated with dietary patterns, indicating that younger older adult individuals have healthier dietary patterns compared to older adult individuals. Education level, sufficiency of income, and occupation before age 60 are significantly positively correlated with dietary patterns. Older adult individuals with higher education levels, sufficient sources of income, or non-agricultural occupations before the age of 60 exhibit healthier dietary patterns. However, participation in health insurance is significantly negatively correlated with dietary patterns, indicating that older adult individuals who participate in health insurance exhibit more healthy dietary patterns compared to those who do not. The study finds no significant effects of place of residence, ethnicity, marital status, or physical exercise on dietary patterns among the older adult.

### 3.4 Quantile regression model

Quantile regression (QR) is a non-parametric regression method that estimates the conditional distribution of the dependent variable at different quantiles, thereby capturing the relationships between the independent and dependent variables under various distribution conditions. Compared to traditional linear regression, quantile regression does not rely on the assumption of a normal distribution of error terms, making it better suited to uncovering the comprehensive characteristics of the data. For the analysis of dietary patterns in this study, quantile regression is applied to examine the effects of living arrangements across different quantiles of dietary health levels, thereby supplementing the linear regression model’s focus on average effects. This study selects three representative quantiles—25th, 50th, and 75th percentiles—to investigate whether the impact of living arrangements on dietary patterns among the older adult varies across different levels of dietary health.

The analysis of the quantile regression model shows that living arrangements have a significant positive impact on the dietary patterns of older adult individuals, with regression results at all quantiles reaching statistical significance. This indicates that, regardless of the level of dietary patterns, living arrangements can improve the dietary health of older adult individuals to some extent. However, it is noteworthy that the coefficients for living with family and living in an institution show a decreasing trend at higher quantiles (75%). This trend may reflect a diminishing marginal effect of living arrangements on dietary patterns as dietary health levels increase. It suggests that for older adult individuals with relatively healthy dietary patterns, the influence of living arrangements is relatively limited, potentially requiring more personalized interventions. Regarding control variables, gender, age, education level, sufficiency of income, and occupation before age 60 all have significant effects on dietary patterns across the three quantiles, with consistent directions. This further underscores the importance of demographic and socioeconomic characteristics in influencing dietary health. For place of residence and health insurance participation, the results indicate heterogeneity in their effects on dietary patterns at the 75th quantile. Specifically, place of residence exhibits a positive effect at the 75th quantile, reflecting the amplification of urban–rural resource distribution imbalances among groups with higher dietary health levels. Conversely, health insurance participation shows a negative effect at the same quantile, which may be related to the tendency of individuals highly reliant on health insurance to reduce autonomous health management. This suggests that for older adult individuals with high health levels, policy design should focus more on optimizing the synergy between health insurance systems and health behaviors. Additionally, physical exercise has a significant positive impact on dietary patterns at the median quantile (50%), which may be linked to the greater promotive effect of health behaviors (such as exercise) on individuals with moderate dietary health. However, this effect is not significant at other quantiles, indicating that its influence may depend on specific health levels or lifestyles. Ethnicity and marital status have no significant effects on dietary patterns across the three quantiles, suggesting that these factors are not key determinants of dietary patterns in this study.

## 4 Mediation analysis

This section primarily examines the mediating effects of loneliness and anxiety on the relationship between living arrangements and dietary patterns among older adults. The results of the mediation analysis are presented in Table 6. It is generally accepted that if the 95% confidence interval (CI) of the Bootstrap method does not include zero and *p* < 0.05, *p* < 0.05, *p* < 0.05, the mediation effect is significant; otherwise, it is not (35, 36, 71). After controlling for demographic and other characteristic variables, the indirect effects of living arrangements on dietary patterns, mediated by loneliness and anxiety, have 95% confidence intervals of [0.0076, 0.0164] and [0.0013, 0.0065], respectively. Both intervals do not include zero, and *p* < 0.05, *p* < 0.05, *p* < 0.05, indicating the presence of significant mediating effects of loneliness and anxiety in the relationship between living arrangements and dietary patterns. This suggests that living arrangements directly influence dietary patterns and also have indirect effects through the “loneliness–dietary patterns” and “anxiety–dietary patterns” pathways. In terms of specific effect sizes, the indirect effect coefficient (a × b) for loneliness is 0.0117, while that for anxiety is 0.0037. According to Cohen’s guidelines (small effect: <0.01; medium effect: 0.01–0.09; large effect: >0.09), the mediating effect of loneliness on the relationship between living arrangements and dietary patterns reaches a “medium effect” level, while anxiety represents a “small effect.” This indicates that although both mediation pathways are statistically significant, the mediating effect of loneliness is notably stronger in magnitude (68, 72).

**Table 6:** Mediation analysis: regression and bootstrap test results for anxiety and loneliness as mediators (*N* = 6,223).

Model/path	Regression coefficient (β\betaβ)	Boot SE	*p*-value	95% CI	*R* ^2^
A. Anxiety as mediator					
M1: Y = Dietary Patterns					
Total Effect (c): X → Y	0.7926	0.0874	0.000	[0.6212, 0.9641]	0.0553
M2: M = Anxiety					
Path (a): X → M	0.2719	0.0878	0.000	[0.0999, 0.4440]	0.0553
M3: Y = Dietary Patterns (X, M)					
Direct Effect (c′): X → Y	0.7661	0.0871	0.000	[0.5954, 0.9369]	0.0851
Path (b): M → Y	0.0974	0.0126	0.000	[0.0728, 0.1221]	0.0851
Indirect Effect (a × b)	0.0037	0.0013	0.000	[0.0013, 0.0065]	–
B. Loneliness as mediator					
M1: Y = Dietary Patterns					
Total Effect (c): X → Y	0.7926	0.0874	0.000	[0.6212, 0.9641]	0.0762
M2: M = Loneliness					
Path (a): X → M	0.3655	0.0297	0.000	[0.3072, 0.4238]	0.0669
M3: Y = Dietary Patterns (X, M)					
Direct Effect (c′): X → Y	0.7089	0.0882	0.000	[0.5359, 0.8819]	0.0818
Path (b): M → Y	0.2292	0.0372	0.000	[0.1563, 0.3021]	0.0818
Indirect Effect (a × b)	0.0117	0.0023	0.000	[0.0076, 0.0164]	–

X represents “Living Arrangements,” MMM represents “Anxiety” or “Loneliness,” and YYY represents “Dietary Patterns”; Regression coefficients are unstandardized and controlled for other relevant variables (e.g., gender, age, education level, etc.); R2 represents the coefficient of determination for each regression equation. Since the indirect effect (a × b) does not constitute an independent regression model, its *R*^2^ value is not reported. The 95% confidence intervals (CIs) are Bias-corrected Bootstrap CIs; Significance levels: *p < 0.05, *p* < 0.05, *p* < 0.05; **p < 0.01, *p* < 0.01, *p* < 0.01; **p < 0.001, *p* < 0.001, *p* < 0.001.

## 5 Conclusion

Under the Healthy Aging Strategy, improving the physical and mental health of older adults has become a critical issue of the era. This study utilizes data from the 2018 Chinese Longitudinal Healthy Longevity Survey (CLHLS) and employs SPSS 26.0 for statistical analysis. Linear regression and bootstrap mediation effect tests are used to explore the relationship between living arrangements and dietary patterns among older adults, as well as the mediating roles of loneliness and anxiety in this relationship. The results reveal the following findings: (1) Living arrangements have a significant positive impact on the dietary patterns of older adults, with loneliness and anxiety serving as partial mediators; (2) Younger older adults generally exhibit healthier dietary patterns compared to their older counterparts. Male older adults have healthier dietary patterns than females. Additionally, education level, income source, and pre-retirement occupation are significantly positively associated with healthier dietary patterns, while participation in medical insurance is negatively associated. Physical exercise shows no significant impact on dietary patterns. These findings suggest that living arrangements positively influence the dietary patterns of older adults by reducing loneliness and anxiety. Further analysis indicates that the mechanism through which living arrangements affect dietary patterns varies depending on individual characteristics such as gender, age, education level, and economic status. These results provide valuable empirical evidence for implementing the Healthy Aging Strategy.

## 6 Discussion and policy recommendations

The impact of living arrangements on dietary patterns through the reduction of loneliness and anxiety is highly significant. Older adults living alone often lack adequate family support and have limited interaction and communication, leaving their material and emotional needs unmet. This makes them more susceptible to psychological issues such as loneliness and anxiety (62, 72, 73). These feelings can further lead to unhealthy eating behaviors (5), resulting in poor dietary patterns (23), characterized by excessive consumption of unhealthy foods like sweets and insufficient intake of healthy foods. In contrast, older adults living with family members benefit from greater emotional support, care, and companionship, which fulfill their needs for security and belonging and significantly reduce their feelings of loneliness (72, 73). Meanwhile, older adults residing in care institutions, although they may face certain social challenges, often benefit from organized social activities and community environments that help alleviate loneliness and anxiety. For instance, nursing homes frequently organize various social events and provide psychological counseling services to help residents build new friendships and manage negative emotions such as loneliness and anxiety. Furthermore, care institutions often employ professional nutritionists to provide balanced meals, which, along with social activities, reduce the occurrence of unhealthy dietary patterns among older adults. Younger older adults tend to have healthier dietary patterns, likely because they are in better physical condition and more mobile, enabling them to adhere to high-quality dietary patterns such as the Mediterranean Diet or the DASH Diet. These dietary patterns emphasize high intake of fruits, vegetables, whole grains, low-fat dairy, fish, and poultry, while reducing sugar, saturated fats, and processed foods. Moreover, compared to women, men are more likely to avoid fried foods, certain meats, and legumes (74, 75). Less healthy dietary patterns are more prevalent among older adults with lower educational attainment (76), as higher education levels are associated with better access to nutritional knowledge and an improved understanding of food nutrition information, leading to healthier dietary choices (41, 77). Individuals with higher education levels are also more proactive in seeking and acquiring nutritional information, which further improves their dietary behaviors (78). Education level is also closely tied to income and occupational status, both of which directly impact dietary patterns. Older adults with higher educational and occupational status tend to have higher incomes and better living conditions. Higher-income individuals generally have healthier dietary patterns (13), as economic resources are strongly associated with access to diverse, high-quality, and nutritious foods, particularly fruits and vegetables (41, 79). Conversely, low-income groups face more constraints in food access, making them more likely to consume high-calorie, low-nutrient foods (80). They are also more vulnerable to rising food prices (11, 81). Finally, the study did not find a significant association between participation in medical insurance and healthier dietary patterns. This may be because medical insurance primarily impacts healthcare costs rather than directly influencing dietary behavior. Additionally, the study found no significant relationship between physical exercise and dietary patterns among older adults. This could be due to the physiological mechanisms by which exercise impacts health rather than directly affecting dietary behavior. Furthermore, the small number of older adults participating in physical exercise in the sample may limit the ability to fully reveal the relationship between physical activity and dietary patterns.

Based on the study’s findings, the following recommendations are proposed: (1) At the Community Level, As one of the primary venues for daily activities among older adults, communities should provide targeted health services tailored to their needs: ① Provide tiered health management services. Develop specialized health management services for older adults with varying health conditions. Establish dedicated health service rooms to offer psychological counseling for older adults with mental health issues and rehabilitation services, including dietary and nutritional guidance, for those with treatable physical conditions. ② Enrich social and recreational activities in the community. Create leisure activity areas for older adults to encourage active participation in community events. Studies indicate that greater involvement in social and cognitive leisure activities helps older adults maintain a positive mindset, which in turn fosters healthier dietary behaviors (82). ③ Enhance digital skills training and learning opportunities. Establish “Senior Universities” or activity centers in communities to provide basic training in digital technologies such as smartphone use. This not only facilitates online interactions with friends and family but also enriches older adults’ entertainment options and strengthens their sense of social inclusion, thereby alleviating loneliness and anxiety to some extent. ④ Promote the implementation of home-based care services. Aging, physical functional decline, and diminished familial roles pose challenges for older adults in adapting to their later years, which can lead to negative emotions such as anxiety, affecting their physical and mental health. Research shows that home-based care reduces the likelihood of anxiety and depression among older adults (83). Social workers or community volunteers should conduct regular home visits for home-based older adults, offering services such as assistance with household chores, medical escort, and companionship to mitigate the emotional and practical challenges faced by “empty-nest” older adult individuals. (2) At the Government Policy Level: ① Promote equitable access to basic public services in urban and rural areas. The government should increase investment in rural community infrastructure and improve public services in healthcare, culture, and recreation to enrich the spiritual and cultural lives of older adults in rural areas. ② Increase subsidies and support for economically disadvantaged older adults. Provide targeted financial subsidies or price support for low-income older adults to enhance the affordability of healthy dietary options. Encourage reemployment among healthy older adults to alleviate financial burdens and improve dietary quality. ③ Strengthen the policy support system. Promote collaborative efforts across healthcare, education, and social security sectors to provide older adults with sustained and comprehensive support. This integrated approach would help older adults maintain stable economic conditions and overall health.

## Data Availability

Publicly available datasets were analyzed in this study. This data can be found here: the 2018 Chinese Longitudinal Healthy Longevity Survey (CLHLS).
